# Multiscale assessment of conservation management for *Amorpha fruticosa* invasion in a marsh meadow

**DOI:** 10.1186/s12862-025-02438-z

**Published:** 2025-09-30

**Authors:** Krisztina Napsugár Nagy, Csilla Evelin Károlyi, László Bakacsy

**Affiliations:** 1 Doctoral School of Environmental Sciences, University of Szeged, Rerrich Béla tér 1, Szeged, 6720 Hungary; 2 Department of Plant Biology, Institute of Biology, Faculty of Science and Informatics, University of Szeged, Közép fasor 52, Szeged, 6726 Hungary; 3 Independent Researcher, Wexford, County Wexford Ireland

**Keywords:** *Amorpha fruticosa*, Biodiversity conservation, Community structure, Invasion management, Marshland, Wetland ecology

## Abstract

This study examined the ecological impact of the invasive shrub *Amorpha fruticosa* in marsh meadows and assessed the effectiveness of combined conservation management practices, i.e., mowing and cattle grazing, in on restoring native vegetation. Conducted in the Mártély Landscape Protection Area (Hungary), the research used a multiscale approach to compare treated non-invaded and treated invaded wetland vegetation. Information theory-based diversity metrics were employed to evaluate the impact of *A. fruticosa* on structural complexity and species composition. Results revealed that although *A. fruticosa* can significantly altered plant community structure, the implemented management strategies effectively reduced its impact. The treated invaded vegetation exhibited diversity levels compareable to native marshland communities, suggesting that mowing and grazing contributed to decreasing *A. fruticose* dominance. However, in the absence of untreated control stands, this pattern must be interpreted cautiously, as the observed similarity could be conclusively attributed to the applied management alone. A slight, but non-significant shift in structural diversity was also observed, implying a residual effect of invasion. This study underscores the value of active, combined conservation strategies in maintaining biodiversity and ecosystem resilience in wetland habitats. The results contribute to broader discussions on invasive species control, emphasizing the role of traditional land-use practices in mitigating the ecological effects of biological invasions.

## Introduction

Invasive plant species pose an increasing threat to wetland ecosystems by disrupting native biodiversity, altering habitat structure, and impairing key ecological functions. Marsh meadows are especially vulnerable due to their dynamic hydrology, species-rich vegetation, and history of anthropogenic disturbance. Among the most severe impacts of plant invasions are reduced species richness, trophic simplification, and breakdowns of spatial patterns vital for ecological resilience [1–3].

A particularly aggressive invader in Central and Eastern Europe is the North American-native woody shrub *Amorpha fruticosa* L. (false indigo bush). It is rapidly spreading through riparian habitats, oxbow lakes, and floodplains, forming dense thickets that suppress native vegetation [4]. Its invasive success stems from traits such as drought and flood tolerance [5], prolific seed production, vegetative sprouting [4], and strong allelopathic properties [6–8]. Prior research has revealed its ability to alter soil pH and nitrogen levels while reshaping microbial communities, further hindering native plant establishment [9]. These changes go beyond species turnover and disrupt plant community structure and spatial organization, increasing ecosystem vulnerability to further invasion and collapse. In particular, they reduce structural complexity, defined as the richness and spatial variability of species combinations across scales [10, 11]. *Amorpha fruticosa* invasion not only lowers plant diversity but also degrades habitat quality for associated fauna, especially marshland birds that depend on open, structurally diverse vegetation for nesting and foraging [12, 13]. Its leaf litter and shading inhibit regeneration of marshland herbs and sedges. Additionally, the shrub’s biochemical exudates, primarily flavonoids and phenolic compounds, can persist in the soil, and delaying native recolonization long after mechanical removal [4, 14]. These traits make *A. fruticosa* a high-priority target for conservation action [10, 11].

To address such invasions, several management strategies have been proposed, including mechanical removal, herbicide application, and active ecological restoration [15, 16]. However, in wetland systems, however, traditional practices such as grazing and mowing, have proven ecologically viable [10, 17]. These methods reduce the biomass of invasive species while promoting microhabitat heterogeneity and native seedling growth [18, 19]. Nevertheless, their effectiveness in restoring spatial structural integrity, rather than just species composition, remains understudied [20, 21].

Most invasion management studies have relied on compositional metrics, such as species richness and Shannon diversity [20, 22]. Although informative, such indices often fail to capture the fine-scale spatial distribution of species, which supports functional interactions, competitive balance, and resilience to disturbance [21, 23]. Structural metrics, including compositional diversity (CD) and the number of realized species combinations (NRC), derived from information theory, offer finer resolution of plant community are organization across spatial scales [24–26]. These tools allow researchers to move beyond floristic inventories and assess how communities reassemble after disturbance or management. In the present study, diversity refers specifically to CD, which captures heterogeneity and complexity of species co-occurrence patterns across spatial scales. Organization refers to the spatial arrangement and patterning of species combinations along the transects, reflecting assembly responses to invasion and management. Structural complexity refers to both species richness and variation in species combinations, measured via CD and NRC. Collectively, these metrics provide a scale-sensitive understanding of vegetation structure beyond conventional diversity indices.

Despite the known ecological impacts of *A. fruticosa*, little is known about how its presence, or removal through management, affects spatial vegetation patterns in marsh meadows. The combined effects of mowing and grazing, although widely used in conservation, have rarely been assessed from a structural perspective. This knowledge gap limits the development of evidence-based strategies for managing wetland invasions. This study investigated how combined conservation practices, comprising mowing and grazing, mitigate the impact of *A. fruticosa* invasion on marsh meadow structural complexity. Using a multiscale spatial framework, we addressed the following questions: (1) How does vegetation diversity differ between managed but non-invaded and *A. fruticosa*-invaded stands? (2) How does *A. fruticosa* influence or distort the structural complexity in plant communities? (3) At what spatial scale are diversity differences become most pronounced between invaded and non-invaded stands?

Using information-theoretic models (CD and NRC functions) applied to high-resolution field data, we explored how management practices affects not only species presence but also the structural fabric of ecological communities. This perspective is key to conservation strategies aimed at reducing invasive biomass while restoring wetland ecosystem function.

## Materials and methods

### Study site

**Fig. 1:**
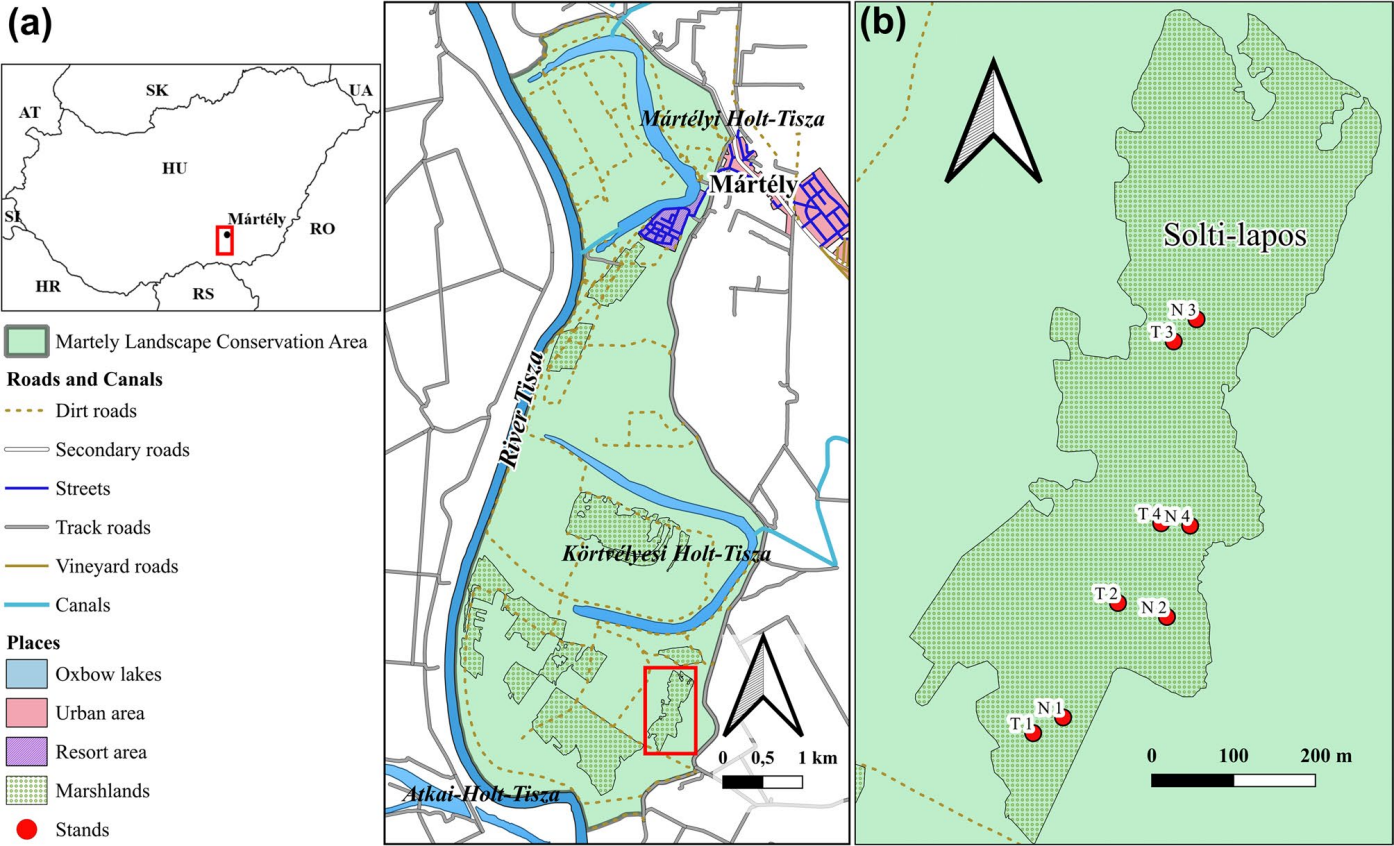
Location and layout of the study area. (**a**) The Mártély Landscape Protection Area, highlighting the Solti-lapos marshland. (**b**) Four pairs of selected stands in the Solti-lapos marshland: N: non-invaded, combined managed stands (1–4), T: *Amorpha fruticosa*–invaded, combined-managed stands

The research site was located within the Mártély Landscape Protection Area of Kiskunság National Park in Hungary (Fig. 1), on the left bank of the Tisza River within its floodplain. The area has been under protection since 1971 and part of the Ramsar Convention since 1979. It encompasses the Körtvélyes and Mártély (Ányási) oxbow lakes, formed by the truncation of two Tisza meanders (Fig. 1a) [27].

The study area includes floodplain marsh meadows and oxbow lake habitats. Marsh vegetation is dominated by typical wet-meadow species, including *Agrostis stolonifera* L., *Phalaris arundinacea* L., and *Alopecurus pratensis* L. The broader landscape contains floodplain forests and plantations dominated by willow and poplar species, which were not part of the study [28–30]. The flora of the oxbow lakes is characterized by species such as *Trapa natans* L., *Salvinia natans* (L.) All., *Hydrocharis morsus-ranae* L., and various *Potamogeton* species [28].

A multiscale study of *A. fruticosa* invasion was conducted in the Solti-lapos marsh meadow (Fig. 1b). This 27.92-ha area was used for both pasture and arable farming until the political transition in 1989. After the transition, conservation-focused management, particularly targeting invasive species was initiated. In 2002, the National Park Directorate launched restoration efforts under the KEOP project, targeting *A. fruticosa*-invaded sites, including Solti-lapos. By the late 2000 s, the land was leased to tenants, and from 2014 onward, lease agreements required the removal of alien woody species (e.g., shrubs and trees) from overgrown former grassland for grassland restoration. At Solti-lapos, management combined grazing and mowing. On average, 25–30 Hungarian Grey Cattle graze the site annually (0.90–1.07 animals/ha), rotating among up to three subareas. Grazing was not continous throughout the grazing season; higher-quality sections were grazed after mowing, whereas lower-quality areas were mown postgrazing for cleanup [31].

Clearance of heavily wooded or *A. fruticosa*-dominated areas occurred in small sections annually or gradually. Annual flooding patterns also influenced the planning and execution of these efforts. The National Park has implemented a conservation strategy that involving the preservation of sedge- and tussock-dominated areas during winter as refuges for overwintering birds, managing in spring, provided they are not inundated [31]. The study area primarily consisted of sensitive permanent grasslands, mainly marsh meadows, classified under the MePAR system [30] (Fig. 1b). This grassland ecosystem was the focus of the study.

### Field sampling

This investigation was conducted in July 2023, during the vegetation period, after the main mowing phase and before postmowing grazing began in most areas. Four vegetation stands were designated as “non-invaded” (N), referring to native marshland unaffected by *A. fruticosa*, and four as “treated” (T), referring to formerly invaded stands where *A. fruticosa* remained present during sampling. In T stands, *A. fruticosa* occurred in 16.7–19.8% of microquadrats, a high frequency given its size and structure. All stands underwent the same mowing and grazing regime; thus, the N-T distinction reflects invasion status only, not treatment history. Stands were paired (N-T), to ensure similar environmental conditions and site histories [32].

Stands were selected randomly while deliberately excluding areas with major disturbance, such as pathways, fallen trees, and animal burrows. A 26-m-long belt transect was established in each stand for sampling. Each transect followed a circular layout, beginning and ending at the same point [23, 33]. Transects comprised 520 sampling subunits, designated as microquadrats, measuring 5 × 5 cm [11, 34]. The 26-m length ensured inclusion of 520 microquadrats, an optimal sample size for information-theoretic structural analysis based on the JNP model [24, 26, 34, 35]. This length also aligned with the average size of homogeneous vegetation patches in the area, enabling high-resolution structural analysis with minimal edge effects. Transects were strategically positioned within the stands to ensure uniform vegetation coverage [26]. Species presence was recorded in each 5 × 5 cm microquadrats along the transects, using Király [36] as the reference for plant identification. Plant material was identified by Krisztina Napsugár Nagy and László Bakacsy. No plant samples were collected, as the sites’s protected status and the noninvasive methodology precluded material remloval.

### Data analyses

The study employed a multiscale analytical framework based on the spatial series methodology developed by Juhász-Nagy [37] and Juhász-Nagy and Podani [24], hereinafter referred to as the JNP model. This approach calculates information-theoretical diversity metrics across hierarchical spatial scales. Specifically, 5 × 5 cm quadrats along 26-m circular transects were aggregated by doubling plot sizes, enabling assessment of diversity and structural complexity at increasing spatial extents [34]. This spatial scaling captures fine-scale beta diversity and community organization, enabling the identification of scales at which vegetation structure is most pronounced. This approach reveals the spatial embedding of species coexistence patterns and highlights scale-dependent effects of invasion [21]. These JNP models include several community-level functions; two were used in this study.

CD reflects the structural diversity of plant communities based on the Shannon diversity of species combinations at each spatial scale. This metric evaluates community complexity based on the frequency distribution of individual species and captures variability in coexistence patterns using information theory. CD values are expressed in binary digits (bits), with smaller quadrat sizes are particularly sensitive to microscale changes in coexistence. Higher CD values reflect greater heterogeneity in species composition and more complex coexistence patterns at the examined spatial scale [25, 26, 37, 38].

NRC quantifies the structural complexity and variability in species coexistence. It indicates how the spatial distribution and abundance of individual species contribute to community pattern formation. NRC is a dimensionless metric that increases exponentially with species richness, making it highly sensitive to such changes. Its maximum values are especially useful for assessing fine-scale beta diversity and estimating landscape-scale variation, particularly when interpreting complex spatial relationships [26, 38, 39]. NRC also enhances understanding of community structure and dynamics through spatial scaling and random reference analysis [21, 25].

High CD values indicate greater compositional diversity, i.e., more heterogeneous species combinations across spatial scales, whereas low values reflect more homogeneous community structure. Similarly, high NRC values suggest more complex coexistence patterns with numerous unique combinations, whereas low values indicate reduced structural variability. All indices were calculated at the transect level using the complete set of 520 microquadrats per stand, thereby reflecting aggregated spatial patterns across scales rather than quadrat-level diversity [21, 34, 38].

In addition to JNP function maxima, the characteristic area (CA) was considered, defined as the spatial scale (in cm²) at which a function reaches its peak [21, 23, 38]. The CA and the function maximum values provide insights into the internal structure and diversity of plant communities. When coexistence is not limited by biotic interactions or environmental filters, species tend to assembly, often producing peak function values at the smallest scales.

The use of CD and NRC functions in field studies to investigate vegetation pattern relationships has been further developed within the INFOTHEM program. JNP function calculation were performed usinf INFOTHEM 3.01 [26, 40], which enables precise scaling of structural diversity during analysis [26, 40, 41]. Species with an occurrence frequency below 2% (present fewer than 11 of 520 quadrats per transect) were excluded to reduce bias and avoid complications from stochastic rare species [22, 42].

To assess the contribution of invasive species to vegetation structure, we analyzed two datasets: one including (T + *Am*) and one excluding (T –*Am*) invasive *A. fruticosa* [43], treating it as equivalent to native species in the former and excluding it from the model in the latter. This approach tested the hypothesis that *A. fruticosa* introduces artificial structural complexity into the invaded community. By removing the dominant invasive species (T –*Am*), we aimed to determine whether observed diversity reflects genuine community structure or is an artifact of invasion. Although both datasets originate from the same plots, their comparison clarifies the structural contribution of *A. fruticosa*. This distinction helps differentiate communities that are intrinsically diverse from those where diversity is inflated by a single dominant species.

Random reference datasets were generated using complete randomization with 5,000 Monte Carlo-generated iterations [24, 26, 44]. This process involved randomly redistributing the occurrence data of all species along each transect [26, 33, 35]. In INFOTHEM, all JNP functions values were normalized at each spatial step based on the number of sampling unit, eliminating bias from sample size differences across scales [26, 40].

### Statistical analyses

A paired *t*-test was used to compare the species frequency of occurrence values, and mixed-effects models were implemented evaluate JNP function values and their CA distribution across stands. Data normality was tested using the Shapiro–Wilks test. Where normality was confirmed, Tukey’s post hoc test was applied; for non-normal data, the, Friedman’s test was used. Statistical significance was set at *p* ≤ 0.05, and results are presented as means ± SD. All statistical analyses and plots were conducted using GraphPad Prism 8.0.1.244 (GraphPad Software, La Jolla, California, USA).

## Results

A comparison of species composition and abundance between transects from the two vegetation types (N and T) revealed no significant differences, except for the presence of *A. fruticosa*. The species recorded were characteristic of the native marsh vegetation community (Table 1).

**Table 1:** Species presence and abundance in transects comprising 520 micro quadrats (5 cm × 5 cm). Analysis was conducted across four pairs of selected stands in the Solti-lapos marshland

Stands Species	1 *N*	1 T	2 *N*	2 T	3 *N*	3 T	4 *N*	4 T	*p*-value (Two-tailed t-test)	
*Amorpha fruticosa*	0	87	0	103	0	57	0	44	0.012	*
*Abietinella abietina*	178	0	294	144	14	0	0	0	0.25	ns
*Agrostis stolonifera*	0	0	11	167	0	0	228	94	> 0.999	ns
*Alopecurus aequalis*	0	168	18	16	0	196	35	56	0.153	ns
*Ballota nigra*	11	0	0	26	23	0	14	0	0.875	ns
*Carex hirta*	187	0	0	0	0	0	0	0	> 0.999	ns
*Carex melanostachya*	0	207	67	324	281	280	100	121	0.159	ns
*Euphorbia lucida*	0	0	12	0	0	0	0	0	> 0.999	ns
*Fraxinus pennsylvanica*	18	0	0	23	11	0	0	0	> 0.999	ns
*Glycyrrhiza echinata*	11	0	30	0	0	20	0	0	0.75	ns
*Iris pseudacorus*	22	0	16	13	14	11	17	10	0.148	ns
*Lycopus europaeus*	0	0	0	0	34	0	14	0	0.5	ns
*Lysimachia vulgaris*	31	12	43	11	21	15	21	14	0.078	ns
*Mentha longifolia*	0	0	0	0	0	0	20	0	> 0.999	ns
*Persicaria hydropiper*	0	0	0	0	0	0	24	0	> 0.999	ns
*Phalaris arundinacea*	107	0	173	0	0	0	33	0	0.25	ns
*Potentilla reptans*	67	43	160	21	0	0	26	53	0.42	ns
*Ranunculus acer*	0	0	0	0	0	0	14	0	> 0.999	ns
*Rubus caesius*	0	11	0	0	0	0	0	0	> 0.999	ns
*Symphytum officinale*	0	0	0	0	23	0	0	0	> 0.999	ns
Species number	9	6	10	10	8	6	12	7	0.095	ns

*N* non-invaded, combined-managed stands (1–4); *T* *Amorpha fruticosa*–invaded, combined-managed stands. Species occuring in less than 2% of the microquadrats in each transect were excluded from the table. Paired *t*-tests (two-tailed) were used for cmoparisons, with significance level was set at *p* = 0.05 (*n* = 4). The significance level is indicated by asterisks (*p < 0.05)

**Fig. 2:**
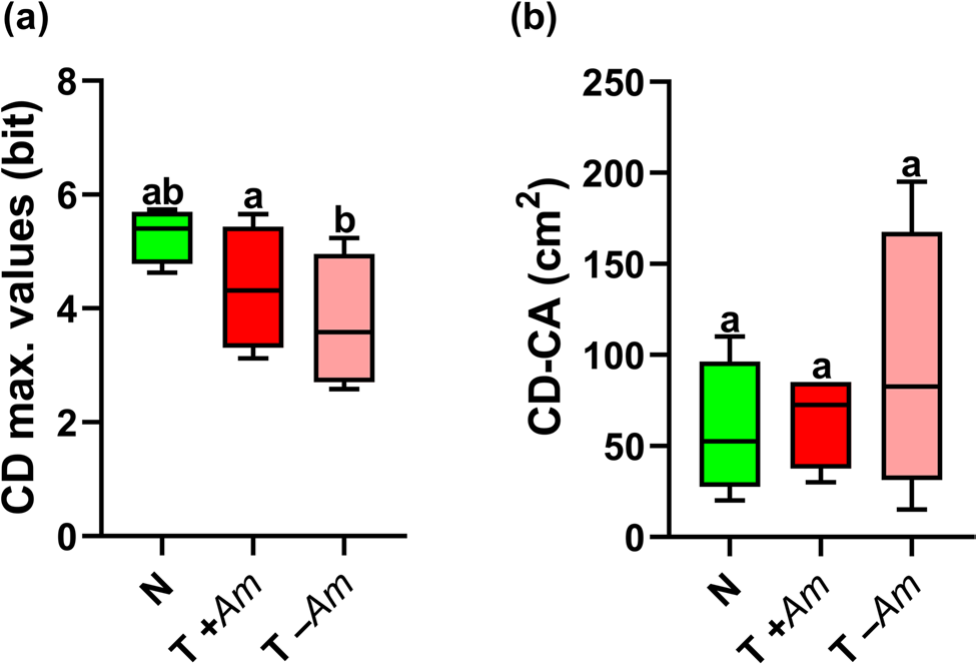
Effects of *Amorpha fruticosa* invasion and combined conservation management on (**a**) compositional diversity (CD) and (**b**) its characteristic area (CD-CA) in marsh meadow. N: non-invaded; T + *Am* - *A. fruticosa* present; T –*Am* - *A. fruticosa* present but excluded from the analysis. For normally distributed data, a mixed effects model (Tukey’s test) was applied; for non-normally distributed data, the Friedman test was used. The least significance level was set at *p* = 0.05 (*n* = 4)

Analysis of the two JNP function parameters showed clear differences between invaded and non-invaded vegetation. Maximum CD values quantifying species combination diversity along transects, were 5.295 ± 0.489 bits in non-invaded stands (N), 4.354 ± 1.102 bits in invaded stands including *A. fruticosa* (T + *Am*), and 3.749 ± 1.182 bits in the same stands with the invasive species excluded (T –*Am*). A significant difference (*p* = 0.01) was found between T + *Am* and T –*Am* vegetation types (Fig. 2). The CA of the CD function was 58.75 ± 37. 5 cm × 5 cm for N vegetation, 65 ± 26.14 cm × 5 cm for T + *Am* stands, and 93.75 ± 74.65 cm × 5 cm for T –*Am* stand pairs. However, no statistically significant differences were observed between the N and T + *Am* (*p* = 0.954), N and T –*Am* (*p* = 0.496), and T + *Am* and T –*Am* (*p* = 0.612) groups (Fig. 2).

**Fig. 3:**
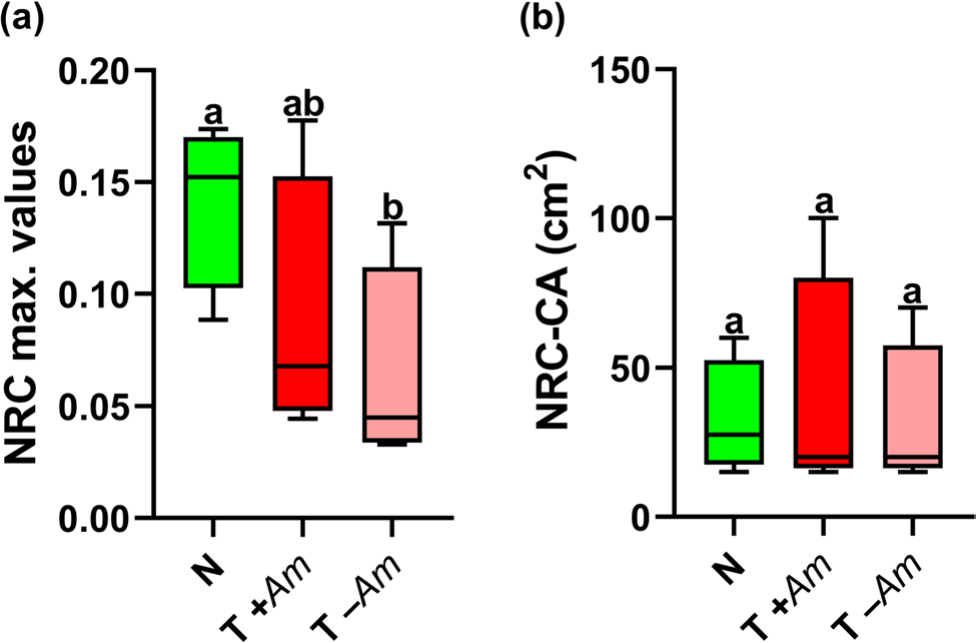
Effects of *Amorpha fruticosa* invasion and combined conservation management on (**a**) the number of realized species combinations (NRC) and (**b**) its characteristic area (NRC-CA) in marsh meadow vegetation. N: non-invaded; T + *Am*: *A. fruticosa* present; T –*Am*: *A. fruticosa* present but excluded from the analysis. For normally distributed data, a mixed-effects model (Tukey’s test) was applied; for non-normally distributed data, the Friedman test was used. The significance level was set at *p* = 0.05 (*n* = 4)

The maximum values of the NRC function were 0.141 ± 0.037 in N vegetation, 0.089 ± 0.060 in T + *Am* stands, and 0.063 ± 0.046 in T –*Am* stands. A significant difference was observed between N and T –*Am* pairs (*p* = 0.039; Fig. 3). The CA of the NRC function was 32.5 ± 19.36 cm × 5 cm for N stands, 38.75 ± 40.9 cm × 5 cm for T + *Am* stand pairs, and 31.25 ± 25.94 cm × 5 cm for T –*Am* stand pairs. However, no significant differences were found between any of the vegetation type pairs (*p* > 0.999 for all comparisons; Fig. 3).

## Discussion

Despite the conservation challenges posed by the *A. fruticosa*, limited research has assessed the effectiveness of its management on community structure and complexity. This study employed a multiscale approach in a marshland vegetation to compare stands invaded by this species, managed by mowing and grazing in combination, with non-invaded, similarly managed natural stands within the Mártély Landscape Protection Area.

Based on prior studies, we hypothesized that combined treatment would not fully mitigate the *A. fruticosa*‘s ecological impact. However, our finding showed no significant differences between N and T pairs in species number or abundance. Although earlier work reported compositional changes in treated invaded vegetation [10, 19, 39], our study showed the opposite outcome. This was based on information-theoretical metrics, CD and NRC, which capture not only species richness but also the structural complexity and spatial patterns of species coexistence. Although sensitive to diversity and coexistence, these indices do not directly detect species turnover. Thus, our findings imply similarity in structural organization, although they do not exclude possible compositional shifts not affecting overall diversity. This pattern likely reflects the mitigation effect of the combined treatment, which may supress *A. fruticosa* dominance through repeated biomass removal, reduce its shading impact on native species and limit allelopathic compounds accumulation. These processes can help preserve native species and maintain structural diversity, even after invasion. Despite past invasion, the treated invaded stands showed a species-rich, invasion-free state (Table 1), indicating that mowing and grazing reduce *A. fruticosa* shading effects, thereby improving light availability for native species. This treatment may also limit the accumulation of allelopathic compounds typically released through continuous leaf fall from the invasive shrub.

The two information theoretic functions characterizing internal structure showed no significant differences in CD or the maximum NRC between N and T + *Am*, stand pairs (Figs. 2 and 3). Although unmowed or untreated stands, were not included in our study, the observed structural similarity between treated natural and invaded stands resembles patterns described by Kun et al. [19], where mowing reduced *Solidago gigantea* Aiton dominance and increased diversity, indicating that active management can mitigate structural homogenization due to invasive species. Despite differing contexts, both studies suggest that conservation management can reverse structural homogenization from invasives. As mowing intensity increased and *S. gigantea* biomass decreased, marsh diversity increased, with marsh vegetation more closely resembling natural, uninvaded conditions. This supports the notion that sustained intervention can progressively restore native structural patterns, even in heavily invaded areas. Similar outcomes were reported by Demeter et al. [10], who examined the impact of cattle grazing on herbaceous and shrub cover and composition in floodplain summer plantations along the Tamiš River, Serbia. They found that continuous moderate-to-intensive cattle grazing suppressed *A. fruticosa* without harming native species.

Our observed outcomes are attributed to the combined treatment’s effect in limiting the ecological impact of *A. fruticosa* on native vegetation, helping preserve the internal structure of native communities. However, the uniform treatment application across all stands makes it difficult to separate the effects of management from those of invasion. This outcome may be due to *A. fruticosa* exerting weaker shading pressure and lower allelopathic material release from continuous leaf drop. Notably, when *A. fruticosa* was excluded from the calculations (T –Am), a weaker but notable difference between natural and invaded treated stands becames apparent (Fig. 2). This suggest that *A. fruticosa* contributes disproportionately to the spatial structure and perceived diversity of the invaded stands. Comparing T + *Am* and T –*Am* is useful for determining how much structural complexity is community-driven versus artificially introduced by the invasive species. This pattern aligns with theoretical expectations from the JNP model: In disturbed or invaded systems, CD typically declines, and CA shifts toward larger spatial scales. This indicates reduced local coexistence and increased spatial homogenization. In contrast, natural or regenerating communities show higher CD and lower CA values, reflecting fine-scale structural complexity and intense local interactions.

Our results suggest that although *A. fruticosa* enhances the apparent spatial structure of invaded communities, it does so primarily through dominance-driven inflation of structural metrics. When its influence is analytically removed, the underlying ecological organization appears degraded. From a management perspective, these findings imply that even when overall species richness remains stable, invasive species can distort the spatial assembly of communities and obscure signs of ecological degradation. Structural metrics, such as CD and NRC, provide a sensitive means of detecting these subtle impacts and reinforced the need for early, sustained suppression of dominant invaders, including *A. fruticosa*. In a related study, Orbán and Bakacsy [42] examined the early-stage of invasion of *Gaillardia pulchella* Foug. in sandy loam grasslands and found substantial variation in CD and NRC values, even when the invasive species was included in the analysis. These results further support the notion that similar species composition and species combinations can persist in the understory of natural wetland vegetation, provided continuous management is maintained.

Contrary to our expectations, the two vegetation types exhibited no significant differences in CA, indicating that marshland vegetation remained organized at the similar spatial scales, regardless of the *A. fruticosa* presence (Figs. 2 and 3). This contrasts with previous studies reporting that invasion often shifts CA values towards larger spatial scales. For instance, Bakacsy [43] found that in sandy grasslands invaded by *Asclepias syriaca* L. CD maxima occurred at broader spatial scales compared with noninvaded stands. This signals a decline in local diversity under the common milkweed compared with natural vegetation. Our findings are also consistent with those of Powell et al. [20], who demonstrated that the effects of floodplain species on native vegetation are structured along characteristic spatial scales that vary widely by species, community type, and management treatment.

The multiscale approach employed in this study demonstrated that cattle grazing supplemented with mowing is a promising *A. fruticosa* management strategy. Nonetheless, further studies involving untreated controls are required to fully assess its independent effectiveness [17], given that the biodiversity in the invaded and treated communities barely differs from that in natural grazed marsh meadows.

## Conclusions

Our findings indicate that combined conservation management practices, specifically mowing and grazing, are effective in mitigating *A. fruticosa* invasion in marsh meadow ecosystems. The structural integrity and species richness of invaded yet managed stands did not differ significantly from those of natural marsh meadows, indicating that these interventions help preserve ecosystem organization. However, subtle structural modifications linked to *A. fruticosa* imply that its presence may still influence long-term community dynamics. The mechanisms driving these effects are likely multifeceted, with grazing and mowing collectively limiting the invasive species’ dominance. Reducted shading and suppression of allelopathic compound accumulation may facilitate native plant species recovery. These results support the hypothesis that continuous, targeted management can maintain biodiversity and stability even in previously invaded habitats. Long-term monitoring would clarify the resilience of these ecosystems and the sustainability of current management practices, helping determine whether such treatments ensure sustained species diversity and structural stability. Future studies should assess alternative or complementary strategies, such as prescribed burning or water level regulation, identify the most effective approaches for long-term invasion control.

## Data Availability

All relevant data are included within the manuscript.
